# The longitudinal influence of cyberbullying victimization on depression, self-esteem and academic burnout among Chinese adolescents: mindfulness as a mediator

**DOI:** 10.3389/fpsyt.2025.1607473

**Published:** 2025-05-08

**Authors:** Yanping Lu, Longxin Dang, Yuan Tao

**Affiliations:** ^1^ 1School of Education Science, Leshan Normal University, Leshan, China; ^2^ School of Nursing, Shandong First Medical University, Tai’an, China; ^3^ Student Mental Health Center, Leshan Normal University, Leshan, China

**Keywords:** cyberbullying victimization, mindfulness, depression, self-esteem, academic burnout

## Abstract

Cyberbullying victimization (CV) has been linked to numerous adverse psychological outcomes among adolescents. While previous research has established associations between CV and negative outcomes including depression, diminished self-esteem, and academic burnout, the potential protective mechanisms in these relationships remain underexplored. This longitudinal study investigated the mediating role of mindfulness in the relationships between CV and these psychological outcomes among Chinese adolescents. A sample of 1,421 Chinese high school students participated in this study. The participants were asked to complete self-report measures of CV, mindfulness, depression, self-esteem, and academic burnout at two time points, approximately six months apart. Structural equation modeling revealed that mindfulness partially mediated the relationship between CV and both depression and academic burnout. Mindfulness fully mediated the relationship between CV and self-esteem. The results highlight the potential value of mindfulness-based interventions in school settings to mitigate the detrimental effects of CV. Limitations of the current investigation and directions for future research and intervention development are discussed.

## Introduction

Digital technologies have fundamentally reshaped social interactions, inadvertently nurturing cyberbullying - characterized distinctively from traditional bullying through anonymity, rapid dissemination, and digital persistence (1). Previous research consistently demonstrates that cyberbullying correlates with diminished psychological well-being and social dysfunction (2). Adolescents remain particularly vulnerable during this developmental period marked by identity exploration and intense peer evaluation (3). The Chinese context presents alarming statistics. A study by Li et al. (4) revealed cyberbullying victimization (CV) rates approaching 31.4%, which highlights the necessity of examining pathways where being cyberbullied impacts youth adjustment. Critically for intervention science, identifying protective mechanisms - particularly mindfulness practices - might buffer these harmful effects. Therefore, this study aims to explore the mediational role of dispositional mindfulness between cyberbullying experiences and psychological outcomes among Chinese adolescents.

This study utilizes the General Aggression Model (GAM; 5) as adapted for cyberbullying by Kowalski et al. (2) as a theoretical framework. The cyberbullying GAM posits that victimization triggers cognitive, affective, and physiological responses, leading to appraisal processes and ultimately various psychological outcomes. This theoretical framework facilitates comprehension of the influence of cyberbullying on depression, self-esteem, and academic burnout, and the potential of mindfulness to modify these pathways.

Cyberbullying represents a significant threat to adolescent well-being, with documented adverse effects across multiple developmental domains. Research has established a clear pattern of associations between CV and numerous indicators of psychological distress, with effects manifesting across diverse cultural contexts. For example, comparative studies have found consistent relationships between cyberbullying experiences and compromised mental health among Swiss, Australian (6) and Japanese adolescents (7). While cross-sectional evidence robustly documents these associations, longitudinal investigations remain comparatively scarce, limiting causal inferences about the temporal stability of cyberbullying effects.

Among the documented psychological consequences, depression emerges as particularly prevalent and concerning. It can represent a key distal outcome within the GAM framework, emerging when cyberbullying triggers sustained negative cognitive and emotional responses. Meta-analytic evidence supports a moderate but consistent association between CV and depressive symptoms (r = 0.24, 95% CI = 0.21-0.27; 2). Prospective research further reinforces this relationship, with longitudinal studies documenting the temporal precedence of cyberbullying to subsequent depressive symptoms among Spanish (8) and British adolescents (9), who exhibited significantly elevated risk for developing depressive symptoms after cyberbullying experiences.

Researchers are paying more attention to self-esteem and academic burnout across research area for cyberbullying among adolescents. Within the GAM framework, self-esteem and academic burnout represent important distal outcomes that may develop when cyberbullying experiences are internalized. During adolescence, young people actively seek out experiences that reinforce positive self-evaluation and the formation of their identity (10). Self-esteem is defined as the degree to which an individual accepts and values themselves, thereby developing fundamental feelings of self-worth (11). It often comes from social support and positive feedback from important people in their lives (12), which is an important part of adolescent development. Academic burnout is when students feel emotionally exhausted from studying, have a detached attitude toward their schoolwork, and feel like their academic abilities are not working well (13). Research shows that depression and burnout often happen together after people are exposed to significant stressors (14, 15). This highlights the importance of looking at what might cause these outcomes after people experience cyberbullying. Studies have found links between being a cyberbullying victim and having low self-esteem and problems in school. In a study of 1,963 American middle school students, Patchin and Hinduja (10) found that cyberbullying victims had much lower self-esteem compared to their non-victimized peers. Research also shows that cyberbullying victims miss more school, do worse in school, and have trouble concentrating (16–18). This suggests that CV has negative effects on how involved students are in school and how well they do. Therefore, it is reasonable that CV may lead to decreased levels of self-esteem and increased levels of academic burnout among adolescents.

Scholarly interest in mindfulness has grown significantly in recent years (19), with emerging research examining its unique relevance for cyberbullying victims (e.g., 20, 21). Mindfulness may function within the GAM as an internal state variable that interrupts automatic negative processing of cyberbullying experiences. Mindfulness - defined as present-moment awareness with non-judgmental acceptance (22) - offers specific mechanisms particularly suited to counteracting cyberbullying effects. First, its decentering component enables victims to observe cyberbullying messages as temporary events rather than reflections of reality, potentially disrupting rumination that maintains psychological distress. Second, the non-judgmental awareness aspect may reduce self-criticism when processing negative online interactions. Third, present-moment focus may counteract the tendency for digital victimization to permeate offline contexts. According to the mindfulness stress-buffering hypothesis (23), these processes collectively modify cognitive-affective responses to cyberbullying stressors, potentially preventing depression (24), preserving self-esteem (25), and maintaining academic engagement (26). These findings suggest that mindfulness may be an important psychological mechanism for adolescents dealing with CV.

Building on the cyberbullying GAM framework introduced earlier (2), we propose that mindfulness operates as a critical internal state that influences how CV impacts psychological outcomes. Specifically, mindfulness may disrupt the typical progression from initial cognitive-affective reactions to sustained psychological distress by altering how victims appraise and process cyberbullying experiences. Empirical evidence supporting this theoretical proposition comes from Royuela-Colomer et al. (21), who demonstrated significant negative associations between mindfulness and CV in a sample of 985 Spanish adolescents. These findings suggest that mindfulness may function as a mediating mechanism through which CV influences depression, self-esteem, and academic burnout - a proposition that aligns with the processing pathways specified in the cyberbullying GAM.

Despite the growing literature on cyberbullying effects, several critical gaps remain. First, while research has established connections between cyberbullying victimization and psychological outcomes, the mechanisms mediating these relationships remain underexplored. Second, most existing studies employ cross-sectional designs, limiting causal inferences about these relationships. Third, few studies have simultaneously examined how mindfulness might differentially mediate effects across multiple outcome domains (emotional, self-evaluative, and academic). Fourth, research on these processes in non-Western contexts, particularly among Chinese adolescents who face unique academic pressures and digital environments, is notably scarce. The present longitudinal investigation addresses these gaps by examining how CV increases depressive symptomatology and academic burnout while decreasing self-esteem through its impact on dispositional mindfulness among Chinese adolescents using a two-wave design spanning six months. Based on the aforementioned literature, we formulated two specific hypotheses: (a) CV would exhibit direct positive associations with depression and academic burnout and negative associations with self-esteem across time; and (b) mindfulness would mediate the relationships between CV and these psychological and academic outcomes.

## Method

### Participants and procedure

Data in this study were drawn from a research program regarding the online behaviors among Chinese children and adolescents. Participants were 1,421 adolescents recruited from six high schools in eastern China using a stratified random sampling approach. At baseline assessment (Wave 1), 31 participants were excluded due to invalid response patterns, yielding an initial analytic sample of 1,390 adolescents (53.8% male; Mage = 15.19 years, SD = 1.52). The sample included students from tenth (31.58%), eleventh (35.64%), and twelfth (32.78%) grades. Among Wave 1 participants, 1,299 consented to follow-up contact, with 1,274 (91.7% of the original sample) completing assessments at Wave 2 approximately six months later. This final longitudinal sample (n = 1,274; 52.8% male; Mage = 15.82 years, SD = 1.37) comprised students across tenth (33.71%), eleventh (36.95%), and twelfth (29.34%) grades. To assess potential attrition bias, we conducted independent samples t-tests comparing completers versus non-completers on all primary study variables. These analyses revealed no statistically significant differences in cyberbullying victimization (t = 0.77, p >.05), mindfulness (t = 0.51, p >.05), depression (t = 1.10, p >.05), self-esteem (t = 0.94, p >.05), or academic burnout (t = 0.88, p >.05), suggesting minimal bias in the longitudinal analyses.

The study protocol received approval from the Ethics Committee of the School of Psychology at the authors’ university. Written informed consent was obtained from both participants and their parents prior to data collection. Questionnaires were administered in quiet classroom settings to randomly selected intact classes within each participating school. Administration occurred during regular school hours under researcher supervision, with most students completing the battery in approximately 15 minutes at both time points. Following questionnaire completion, researchers conducted 20-minute group activities as compensation for participation. Additionally, all participants received informational booklets containing contact information for reputable psychological support services and a curated list of self-help resources.

### Measures

#### Cyberbullying victimization

The Chinese version of the Revised Cyber Bullying Inventory-Cyberbullying Subscale (RCBPI-CS; 27) was used to measure adolescents’ experiences with cyberbullying. This scale evaluates the occurrence and frequency of cyberbullying victimization over a six-month period. This subscale contains 14 items that are rated on a four-point scale from 1 (not at all) to 4 (more than three times). Examples items included “I was insulted by someone online” and “I received false information online to defame me”. This scale was applied to Chinese adolescents with good validity and liability (27). In this study, the scale demonstrated good internal consistency (α = 0.89).

#### Depression

The Center for Epidemiologic Studies Depression Scale for Children (CES-DC) was used to assess depression with a 4-point response option (0 = not at all, 1 = a little, 2 = some, 3 = a lot). The CES-DC was translated into Chinese in the early 1990s and was validated with various Chinese populations (28). Examples of items included “I feel scared” and “I have poor sleep”. Scores on the scale indicate the severity of depression. The internal consistency of the scale in this study was satisfactory (α = 0.81).

#### Self-esteem

Self-esteem was measured using the Chinese version of the Rosenberg Self-Esteem Scale (RSES-C; 29), a 10-item scale developed specifically for the Chinese population. Participants were asked to rate each item on a 4-point Likert scale ranging from 1 (strongly disagree) to 4 (strongly agree). Examples of the items included “I feel that I have a number of good qualities” and “All in all, I am inclined to feel that I am a failure”. Higher scores indicated a higher level of self-esteem. In the present study, the scale demonstrated excellent internal consistency (α = 0.91).

#### Academic burnout

Academic burnout was assess using the Learning Burnout Questionnaire (LBQ). The LBQ is a 21-item scale developed in a Chinese student sample to assess academic burnout (30). Participants were asked to rate each item on a 5-point scale ranging from 0 (not at all) to 4 (almost always). The LBQ includes four subscales: mental exhaustion, the lack of personal learning accomplishment, the alienated relationship between students and teachers, and physical exhaustion. Examples items included: “I don’t care whether I finished my homework” and “I don’t want to study”. Higher scores indicated greater severity of academic burnout. In this sample, the scale demonstrated good internal consistency (α = .86).

#### Mindfulness

Mindfulness was measured using the Chinese version of the Mindfulness Attention Awareness Scale (MAAS; 31). The original MAAS (32) is a widely-used scale to assess an individual’s mindfulness. It contains 15 items, each rated on a 6-point scale from 1 (always) to 6 (never). Examples of the items included “I find myself doing things without paying attention” and “I rush through activities without being attentive to them”. All items were reverse-scored to compute a composite score, with higher scores indicating higher levels of mindfulness. This scale has demonstrated good reliability and construct validity in Chinese adolescent samples (26, 33). In the present study, the scale demonstrated excellent internal consistency (α = 0.91).

### Data analysis

Analyses were conducted using SPSS 23.0 and AMOS 21.0. Preliminary data screening revealed a minimal proportion of missing values (3.84% across all variables). Little’s Missing Completely at Random (MCAR) test indicated that missing data patterns were randomly distributed across all measures (p >.05), supporting the use of full information maximum likelihood estimation. Descriptive statistics were calculated to examine the central tendency and variability of primary study variables. Bivariate relationships among T1 CV, T2 mindfulness, and T2 outcome variables (depression, self-esteem, and academic burnout) were assessed using Pearson’s product-moment correlations.

Structural equation modeling with maximum likelihood estimation was employed to test the hypothesized mediational pathways. The temporal sequencing of variables in our model specified T1 CV as the predictor, T2 mindfulness as the mediator, T2 depression, self-esteem, and academic burnout as outcome variables, and T1 depression, self-esteem, and academic burnout as covariates. Statistical significance of the indirect effects was evaluated using bootstrapping procedures with 5,000 resamples to generate bias-corrected 95% confidence intervals. Model fit was assessed using multiple indices: the ratio of chi-square to degrees of freedom (*χ*²/df), Tucker-Lewis Index (TLI), Comparative Fit Index (CFI), and Root Mean Square Error of Approximation (RMSEA). Following conventional guidelines, values of TLI and CFI exceeding.90, RMSEA below.08, and *χ*²/df below 5 were considered indicative of acceptable model fit.

## Results

We first applied Harman’s single-factor test to examine common method bias (34). All items relevant to the study were subjected to exploratory factor analysis, and the unrotated factor solution was examined to determine the number of factors that are necessary to account for the overall variance. This procedure suggested eight factors, while no single factor accounted for the majority of the covariance among the variables. Therefore, no significant common method bias existed in the current study.

The values of means, standard deviations (SD) and the Pearson’s correlations among all variables included in the model are reported in **Table 1** which shows significant correlations between T1 CV, T2 mindfulness, T2 depression, T2 self-esteem, and T2 academic burnout.

**Table 1:** Means, standard deviations (SD) and correlations of all the variables (N = 1274).

	M ± SD	1	2	3	4	5
1. T1 CV	20.72 ± 5.13	–	.566^***^	-.418^***^	.341^***^	-.502^***^
2. T2 Depression	20.17 ± 11.40	.567^***^	–	-.341^***^	.400^***^	-.495^***^
3. T2 Self-esteem	26.22 ± 8.16	-.426^***^	-.339^***^	–	-.431^***^	.189^**^
4. T2 Academic burnout	41.65 ± 12.64	.332^***^	.402^***^	-.422^***^	–	-.442^***^
5. T2 Mindfulness	60.24 ± 15.15	-.522^***^	-.477^***^	-.184^**^	-.458^***^	–

Left/bottom triangle is the Pearson’s correlations of all the variables, right/top triangle is the partial correlations of all the variables by age, gender, grade and Internet use. ^***^p <.001, ^**^p <.01.

We built a measurement model that included four observed variables and one latent variable. In this measurement model, correlations were specified between CV, mindfulness, depression, self-esteem, and academic burnout. Factor loadings for the manifest indicators on their respective variables were estimated freely. The model was found to fit the data well [*χ^2^/df* = 3.294, CFI = 0.924, TLI = 0.919, RMSEA (90% CI) = 0.068 (0.050 - 0.084)]. The measurement model was sound and suitable for further analysis of the structural equation model.

Based on this measurement model, we then built a direct effect model that demonstrated the effects of T1 CV on depression, self-esteem, and academic burnout at T2. This model also fit the data well [*χ^2^/df* = 2.468, CFI = 0.948, TLI = 0.951, RMSEA (90% CI) = 0.058 (0.045 - 0.077)]. Path analyses revealed that T1 CV was a significant positive predictor of T2 depression (*β* = 0.41, *p* < 0.001) and T2 academic burnout (*β* = 0.28, *p* < 0.001) and T1 CV was also a significant negative predictor of T2 self-esteem (*β* = -0.40, *p* < 0.001).

**Figure 1:**
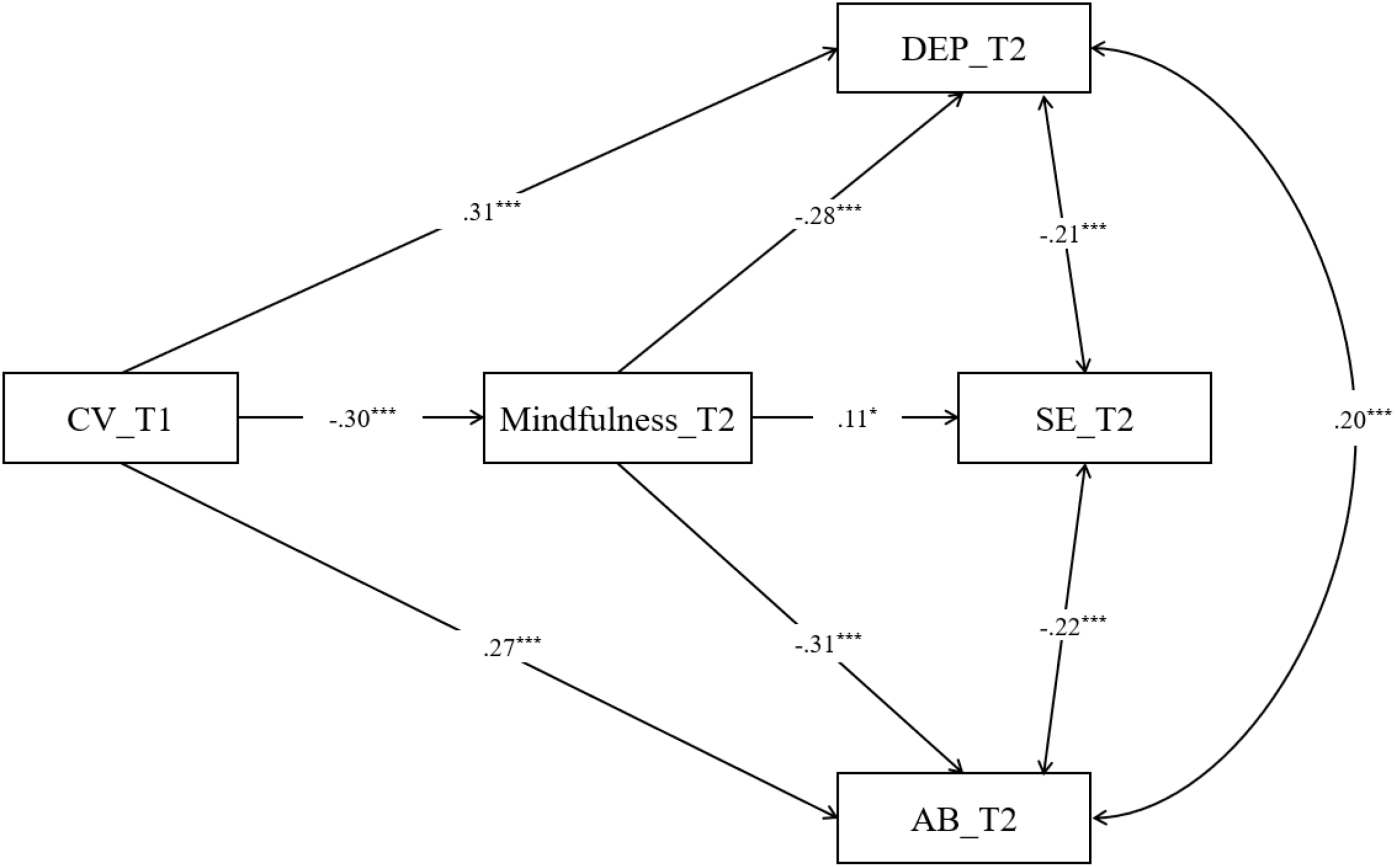
Examined mediation model with standardized beta weights and significance levels added. For clarity, insignificant path coefficient has not been depicted in the figure. CV, cyberbullying victimization; SE, self-esteem; AB, academic burnout; ^***^ *p* < 0.001, ^*^ *p* < 0.05.

Based on the direct effect model, we inserted T2 mindfulness between T1 CV and T2 depression and T2 self-esteem and T2 academic burnout to establish an indirect effect model (see **Figure 1**). We put age, gender, grade and Internet use (time and frequency) into a measurement model as covariate, as they may be associated with cyberbullying among adolescents (8, 35, 36). We found that this indirect effect model also fit the data well [*χ^2^/df* = 1.854, CFI = 0.971, TLI = 0.982, RMSEA (90% CI) = 0.038 (0.027 - 0.054)]. **Figure 1** shows the path coefficients of the model. All path coefficients were statistically significant. More importantly, bootstrap analyses showed that the relationship between T1 CV and T2 depression and T2 self-esteem and T2 academic burnout was mediated by T2 mindfulness. The relation of T1 CV and T2 self-esteem was fully mediated by T2 mindfulness.

Finally, the bias-corrected bootstrap test was conducted to further evaluate the significance and CIs of the mindfulness mediating effects; 5000 bootstrap samples were created from the original data set using random samples with replacement. If the 95% confidence interval for the estimate of the indirect path coefficient does not include 0, it could be concluded that the indirect path coefficient is significant. **Table 2** illustrates these results, which indicate that cyberbullying victimization had an indirect effect on depression, self-esteem and academic burnout through mindfulness.

**Table 2:** Bias-corrected bootstrap test on mediating effects.

Indirect paths	*β*	*B*	Standardized 95% CI	
			Low	High
CV-Mi-DEP	.099^**^	.046	.069	.132
CV-Mi-SE	-.054^*^	-.012	-.072	-.031
CV-Mi-AB	.115^**^	.087	.081	.140

CV, cyberbullying victimization; Mi, mindfulness; DEP, depression; AB, academic burnout; ^**^p <.01, ^*^p <.05.

## Discussion

The present study examined the mediating role of mindfulness in the associations between CV and psychological outcomes (depression, self-esteem, and academic burnout) using a longitudinal design with 1,274 Chinese adolescents. This investigation represents, to our knowledge, the first longitudinal examination of these interrelated constructs. Our findings revealed that mindfulness partially mediated the relationships between CV and both depression and academic burnout, while fully mediating the relationship between CV and self-esteem across the six-month timeframe.

Consistent with our hypothesis and previous research (8, 9), CV positively predicted depression and academic burnout, and negatively predicted self-esteem over time, controlling for demographics and internet use patterns. The prospective association between CV and increased depressive symptoms aligns with developmental psychopathology frameworks, wherein online victimization constitutes a significant stressor during a sensitive developmental period (37). The relationship between adversities and mental health outcomes has been well-documented (e.g., 38–40). The negative association between CV and self-esteem reflects adolescents’ heightened sensitivity to social evaluation (41), with cyberbullying potentially undermining positive self-evaluation by introducing negative feedback in digital contexts. These findings support General Strain Theory (42), positioning cyberbullying as a stressor precipitating psychological distress.

Supporting our second hypothesis, baseline CV negatively predicted mindfulness six months later, consistent with previous research (21, 24). Mindfulness partially mediated the relationships between CV and both depression and academic burnout, suggesting additional pathways exist through which CV influences these outcomes. The full mediation finding regarding self-esteem indicates mindfulness may play a crucial role in maintaining positive self-regard following cyberbullying experiences, allowing adolescents to recognize cyberbullying messages as external events rather than valid reflections of their self-worth (22, 43).

These findings extend the cyberbullying GAM (2) by identifying mindfulness as a key internal state mediating the relationship between cyberbullying encounters and psychological outcomes. CV may diminish adolescents’ capacity for present-moment awareness and non-judgmental acceptance, contributing to psychological distress and reduced academic engagement.

While our findings suggest mindfulness mediates the relationship between cyberbullying victimization and psychological outcomes, alternative explanations warrant consideration. The observed relationship between CV and reduced mindfulness may be influenced by unmeasured intervening processes. For instance, cyberbullying experiences might trigger ruminative thinking patterns, where victims repetitively focus on negative experiences, thereby reducing present-moment awareness characteristic of mindfulness. Similarly, cyberbullying may induce hypervigilance to social threats, with victims continuously scanning for potential danger in both online and offline environments, disrupting mindful attention. Additionally, sleep disturbances following cyberbullying incidents could impair attentional control necessary for mindfulness. The stress-generation hypothesis provides another perspective, suggesting that individuals with lower dispositional mindfulness might engage in behaviors that increase likelihood of subsequent victimization.

Some limitations of this study need to be acknowledged. First, reliance on self-report measures for all constructs introduces potential shared method variance, potentially inflating observed relationships (44). Future studies should incorporate multiple assessment methods, including peer reports of cyberbullying, behavioral assessments of mindfulness, and clinical interviews for depression. Second, while our longitudinal design represents an improvement over cross-sectional approaches, the two-wave design precluded examination of more complex temporal dynamics. Three-wave designs would enable more rigorous testing of mediation processes by separating measurement of predictors, mediators, and outcomes (45). Future research should incorporate multiple assessment methods, employ three-wave designs, examine these relationships across diverse socioeconomic and cultural contexts, account for potential moderators, and explore these processes among cyberbullying perpetrators and bully-victims (46). To specifically address these limitations, we recommend several promising research directions. First, researchers could employ ecological momentary assessment designs to capture real-time responses to cyberbullying incidents, providing more ecologically valid measurements of mindfulness processes. Second, longitudinal growth mixture modeling could identify distinct trajectory patterns of recovery following cyberbullying victimization, potentially revealing for whom mindfulness is most effective. Third, experimental designs comparing different mindfulness components (e.g., focused attention versus acceptance training) could determine which elements most effectively buffer cyberbullying effects. Regarding moderators, future studies should examine: (1) developmental factors such as adolescent cognitive maturation, (2) contextual factors including family communication patterns and school climate, (3) individual difference factors such as trait rumination and emotion regulation capacity, and (4) cyberbullying-specific factors including severity, publicity, and frequency of incidents in determining when mindfulness most effectively mitigates negative outcomes.

Despite these limitations, this study has a few implications. This study adds to our understanding of cyberbullying by combining Kowalski et al.’s (2) cyberbullying model with mindfulness practices. It also expands these theoretical frameworks to Chinese adolescents. Our findings suggest that mindfulness is an important internal state that influences how cyberbullying experiences affect psychological outcomes, especially for how people perceive themselves. From an intervention perspective, our findings suggest that mindfulness-based approaches may help reduce the negative effects of cyberbullying experiences. School-based mindfulness programs have been shown to improve adolescent well-being (47), and they might be especially helpful for those experiencing cyberbullying. Specific mindfulness practices that may be particularly beneficial include brief focused breathing exercises (3–5 minutes) that could be integrated into daily classroom routines, body scan practices to help students recognize stress responses following cyberbullying incidents, and guided self-compassion exercises to counter negative self-perceptions from online harassment. Schools could implement these practices through tiered approaches: universal classroom-based mindfulness for all students (tier 1), small group interventions for at-risk students (tier 2), and targeted individual support for identified cyberbullying victims (tier 3). Integration could occur through existing advisory periods, health curricula, or after-school programs, with digital literacy education incorporating mindfulness components specifically designed for online contexts. Additionally, programs teaching digital literacy could include mindfulness exercises to help adolescents respond to online harassment with more awareness and emotional control.

## Conclusion

In conclusion, this longitudinal investigation provides evidence that mindfulness mediates the relationship between CV and psychological outcomes among Chinese adolescents, highlighting the potential protective role of mindfulness in digital contexts.

## Data Availability

The raw data supporting the conclusions of this article will be made available by the authors, without undue reservation.
